# Persistent Occiput Posterior position - OUTcomes following manual rotation (POP-OUT): study protocol for a randomised controlled trial

**DOI:** 10.1186/s13063-015-0603-7

**Published:** 2015-03-15

**Authors:** Hala Phipps, Jon A Hyett, Sabrina Kuah, John Pardey, Joanne Ludlow, Andrew Bisits, Felicity Park, David Kowalski, Bradley de Vries

**Affiliations:** RPA Women & Babies, Royal Prince Alfred Hospital, Sydney, NSW Australia; Discipline of Obstetrics, Gynaecology and Neonatology, University of Sydney, Sydney, NSW Australia; Women’s and Children’s Hospital, Adelaide, SA Australia; Nepean Hospital, Penrith, NSW Australia; Royal Hospital for Women, Sydney, NSW Australia; The John Hunter Hospital, Newcastle, NSW Australia; Canterbury Hospital, Sydney, NSW Australia

**Keywords:** Posterior position, Caesarean section, Fetal malposition, Occipito-anterior position, Operative delivery, Instrumental delivery, Abdominal ultrasound

## Abstract

**Background:**

Occiput posterior position is the most common malpresentation in labour, contributes to about 18% of emergency caesarean sections and is associated with a high risk of assisted delivery. Caesarean section is now a major contributing factor to maternal mortality and morbidity following childbirth in developed countries. Obstetric intervention by forceps and ventouse delivery is associated with complications to the maternal genital tract and to the neonate, respectively.

There is level 2 evidence that prophylactic manual rotation reduces the caesarean section rate and assisted vaginal delivery. But there has been no adequately powered randomised controlled trial. This is a protocol for a double-blinded, multicentre, randomised controlled clinical trial to define whether this intervention decreases the operative delivery (caesarean section, forceps or vacuum delivery) rate.

**Methods/Design:**

Eligible participants will be (greater than or equal to) 37 weeks’ with a singleton pregnancy and a cephalic presentation in the occiput posterior position on transabdominal ultrasound early in the second stage of labour. Based on a background risk of operative delivery of 68%, then for a reduction to 50%, an alpha value of 0.05 and a beta value of 0.2, 254 participants will need to be enrolled.

This study has been approved by the Ethics Review Committee (RPAH Zone) of the Sydney Local Health District, Sydney, Australia, and protocol number X110410.

Participants with written consent will be randomised to either prophylactic manual rotation or a sham procedure. The primary outcome will be operative delivery (defined as vacuum, forceps and/or caesarean section deliveries). Secondary outcomes will be caesarean section, significant maternal mortality/morbidity and significant perinatal mortality/morbidity.

Analysis will be by intention-to-treat. Primary and secondary outcomes will be compared using a chi-squared test. A logistic regression for the primary outcome will be undertaken to account for potential confounders.

The results of the trial will be presented at one or more medical conferences. The trial will be submitted to peer review journals for consideration for publication. There will be potential to incorporate the results into professional guidelines for obstetricians and midwives.

**Trial registration:**

The Australian New Zealand Clinical Trials Registry ACTRN12612001312831. Trial registered 12 December 2012.

## Background

Persistent occiput posterior (OP) position is associated with 18% of intrapartum caesarean sections and a high risk of assisted vaginal delivery [1-3]. Caesarean section is now a major contributing factor to maternal mortality and morbidity following childbirth in developed countries [4,5]. Obstetric intervention by forceps and ventouse delivery is associated with complications to the maternal genital tract and neonate, respectively [6-8].

Manual rotation from the OP to the occiput anterior (OA) position is a safe, relatively simple and easy to perform procedure that could reduce the operative delivery rate (defined as vacuum delivery, forceps delivery and/or caesarean section) and therefore increase the chances of a normal vaginal birth [9]. It is performed by only a minority of obstetricians and midwives in Australia and New Zealand, yet is considered to be acceptable by the vast majority [10,11]. However, obstetricians and midwives would perform a manual rotation if there was evidence that it reduced the risk of operative delivery to 50% or less [10,11] suggesting that demonstration of efficacy will translate into clinical practice.

Preliminary studies of efficacy are promising, but there has been no adequately powered randomized controlled trial (RCT) [12,13]. It has been recommended that RCTs be conducted to explore the efficacy of manual rotation in the management of OP labours [14].

### Epidemiology

The prevalence of the OP position is 15 to 32% at the onset of labour [15-18], 10 to 20% early in the second stage of labour and 5 to 8% at delivery [2,17,19,20]. The operative delivery rate varies from 54% to 82% when the OP position is present at delivery, compared with 6% to 22% when the fetus is in the more common OA position [3,16,19,20]. When the OP position is present at the beginning of the second stage of labour, the operative delivery rate was about 70% in two higher risk cohorts [2,3].

Thus, of all women who plan to have a normal vaginal birth, 10 to 20% will have a fetus in the OP position early in the second stage of labour. These women will be eligible to have a manual rotation to modify their background risk of up to 70% of obstetric intervention with forceps, vacuum or caesarean section.

### Complications of the occiput posterior position

The OP position is associated with more frequent induction and augmentation of labour and prolonged first and second stage of [3,17,18,21], chorioamnionitis, post-partum haemorrhage, third and fourth degree perineal tears, wound infection and endometritis [22,23]. Associated adverse neonatal outcomes include birth trauma, low 5-minute Apgar score, and admission to the neonatal intensive care unit [24].

### The intervention in current practice

Manual rotation is a well-accepted component of obstetric practice, particularly in the context of rotating the fetus to the OA position immediately prior to the application of non-rotational forceps such as Neville-Barnes [25]. However, it is also used commonly in a prophylactic setting (without assisted delivery) to reduce the complications associated with OP delivery [12,13]. In a survey of obstetricians in Australia and New Zealand, 70% believed it was acceptable in a prophylactic setting; but only 38% had performed a manual rotation in the last year, and most of these had only performed one or two [10]. Both obstetricians and midwives reported they would perform a manual rotation if there was evidence that it would reduce the chances of operative delivery from 68% to 50% or less [10,11]. Thus demonstration of efficacy would provide substantial scope for the intervention to be introduced into widespread practice.

### The efficacy of the intervention

Preliminary cohort studies report that manual rotation is associated with a reduction in caesarean section and adverse maternal outcomes:

1. In a retrospective cohort study, Schaffer *et al.* (2006) (n = 731) reported that the caesarean section rate was lower in women who had a successful manual rotation compared to when the fetus was unable to be rotated (2% versus 34%) [26]. However, there was no control group of women for whom a manual rotation was not performed and it is not possible to know if this was due to the procedure itself or to underlying confounders such as a smaller fetus.
2. In a prospective cohort study with historical controls (n = 61), the local labour ward policy was changed from not performing prophylactic manual rotation to routinely performing the procedure for OP position about ‘half way’ into the second stage of labour [12]. The operative delivery rate for fetuses in the OP position fell from 73% prior to the change in policy to 23% after the policy was implemented, but this study design is subject to a significant risk of bias.
3. Schaffer *et al*. (2011) re-reported their 2006 data with a control group identified retrospectively from a database and found a 9% risk of caesarean section when manual rotation was performed compared with a 41% risk when it was not [13]. However, the authors had information on the fetal position at the time of birth but not earlier in the second stage of labour when the procedure was performed. Thus OP fetuses that were destined to rotate naturally to the OA position would have been included in the intervention group, but not the control group, which would result in an overestimation of the caesarean section rate in the control group and of the efficacy of manual rotation.

Thus preliminary studies suggest that manual rotation reduces the risk of operative delivery but are susceptible to significant bias. An RCT would best provide unbiased answers regarding the effects of manual rotation of the fetal occiput on maternal and perinatal outcomes.

### The safety of the intervention

Manual rotation has long been considered to be safe [9]. One retrospective cohort study reported lower rates of complications when it was performed for OP position compared to when it was not (Table 1) [13]:

**Table 1:** **Complications of manual rotation versus expectant management (Shaffer 2011)** [13 **]**

**Complication**	**Manual rotation (n = 731)**	**Expectant (n = 2,527)**	***P***
Postpartum haemorrhage	22.3%	33.1%	<0.001
3^rd^ and 4^th^ degree tears	15.7%	20.1%	0.017
Cervical laceration	2.2%	1.0%	0.024
Chorioamnionitis	8.6%	14.4%	<0.001
Endometritis	3.6%	7.2%	<0.001
5-min Apgars <7	1.8%	3.7%	0.011
Umbilical cord arterial pH			
<7	0.6%	1.4%	0.15
Base excess < −12	3.5%	3.2%	0.73
Shoulder dystocia	2.1%	1.1%	0.064
Birth trauma	1.09%	1.23%	0.77

Thus, third and fourth degree tears, chorioamnionitis, post-partum haemorrhage, endometritis and 5-minute Apgars less than 7 all improved when prophylactic manual rotation was performed. but cervical laceration was increased.

In the POP-OUT trial manual rotation will be performed at full dilatation, which theoretically will minimize the risk of cervical laceration.

There is also a single case report of an umbilical cord prolapse associated with a manual rotation [27]. In this report, an emergency caesarean section was performed and the baby was born alive and presumably well. Other risk factors such as amniotomy, application of a fetal scalp electrode and external cephalic version were more frequently associated with umbilical cord prolapse [27].

### The timing of the intervention

Manual rotation from the OP position may be performed at full cervical dilatation or late in the first stage of labour. In a French case control study (n = 147) in a labour ward where prophylactic manual rotation was performed routinely, two risk factors for inability to rotate the fetus were identified: [1] attempted rotation before full dilatation and [2] failure to progress in labour [28]. Thus. we consider that it would be reasonable to attempt prophylactic manual rotation after full dilatation is achieved, but relatively early in the second stage of labour, before the fetal head becomes impacted in the maternal pelvis.

### Rationale for operative delivery as the primary outcome

Operative delivery was selected as the primary outcome for the POP-OUT Trial because it is clearly associated with important short- and long-term outcomes for the woman and her baby [6-8,29-32]. Other important obstetric parameters will be measured, but reported as secondary outcomes. Reducing the rate of operative delivery for OP position is perceived to be very important by obstetricians and midwives [10,11]. In high income countries, emergency caesarean section is associated with significant maternal morbidity and a fivefold increase in maternal mortality [33].

### Explanation for choice of comparator

A sham procedure was chosen as a comparator to minimize the risk of performance bias. There would be substantial scope for management to differ according to treatment allocation if it was known. For example, a women could be encouraged to push more strongly if her midwife was aware that a manual rotation had been performed.

## Methods/Design

### Aim

The aim of the study is to determine the efficacy of elective manual rotation in the management of OP position in the second stage of labour.

### Hypothesis

Among women who are at least 37 weeks gestation and whose baby is in the OP position early in the second stage of labour, manual rotation compared with a ‘sham’ rotation will result in a reduction in operative delivery.

### Primary objectives

The primary objectives are to determine the differences between intervention and control groups in the operative delivery rate (defined as vacuum, forceps and/or caesarean section deliveries).

### Secondary objectives

The secondary objectives are to determine the differences between intervention and control groups in caesarean section, in the combined measure of serious maternal morbidity and mortality within six weeks of birth, and in the combined measure of serious perinatal/neonatal morbidity and mortality within six weeks of birth.

### Trial design

The POP-OUT trial is designed as a superiority, double-blinded, multicentre, randomised controlled clinical trial with two parallel groups and a primary endpoint of operative delivery. Randomization will be performed as block randomization with a 1:1 allocation.

### Study settings

Hospitals in Australia that have 2,000 or more deliveries per year include the following:

1. Canterbury Hospital, NSW
2. The John Hunter Hospital, NSW
3. The Nepean Hospital, NSW
4. The Royal Hospital for women, Randwick, NSW
5. The Royal Prince Alfred Hospital, NSW
6. The Women and Children’s Hospital, SA

We do not intend to recruit in any other centres. A list of participating centres may be found at www.popout.me/participating-hospitals.

### Eligibility criteria

#### Inclusion criteria

Inclusion criteria include the following:

1. age ≥ 18 years
2. singleton pregnancy
3. ≥37 weeks of gestation
4. planned vaginal birth
5. cephalic presentation
6. full cervical dilatation
7. occiput posterior position confirmed by ultrasound where the occiput is <45° from the midline

#### Exclusion criteria

Most exclusion criteria were selected on the basis of predisposition to requiring an operative delivery and are as follow:

1. clinical suspicion of cephalopelvic disproportion
2. previous caesarean section
3. brow or face presentation
4. ‘Pathologic’ CTG according to RCOG classification plus either baseline >160 beats per minute or reduced variability
5. fetal scalp pH <7.25 or lactate >4
6. known or suspected chorioamnionitis
7. intrapartum haemorrhage >50 mL
8. temperature ≥38.0°C in labour
9. pre-existing maternal diabetes
10. suspected fetal bleeding disorder (theoretical risks associated with procedures involving manipulation of fetal position)
11. known major anatomical fetal abnormality (could influence safety or efficacy of manual rotation).

### Eligibility criteria for study centres

Ability to provide a 24-hour on-call service with experienced operators to perform the intervention.

### Individuals who will perform the intervention

Only obstetricians or midwives who are experienced in performing a manual rotation and have performed at least 20 procedures will participate in the study. All operators will complete a questionnaire outlining their technique and experience.

### Intervention: manual rotation

#### Intervention description

Manual rotation is performed at full dilatation if the fetal position is OP. The technique employed will be at the discretion of the operator performing the procedure.

With the membranes ruptured, a vaginal examination is performed and the woman is asked to bear down. Constant pressure is exerted with the index finger against the lambdoid suture to rotate fetal head. This may take 2 to 3 contractions and the position is commonly held for two contractions while the woman bears down to reduce the risk of reverting back to the OP position.

Alternatively, the examiner places two fingers behind the fetal ear or the entire hand behind the occiput and applies constant flexion and rotation to the fetal head.

For purposes of the POP-OUT Trial, the procedure will be described as a ‘manual rotation (digital)’ if only the fingers are used and as a ‘manual rotation (whole hand)’ if the whole hand is used.

### Comparator: sham procedure

#### Comparator description

Women randomized to the ‘sham rotation’ will have the same apparent vaginal examination as the intervention but no rotational force will be applied. The woman is asked to bear down. The accoucheur places fingers in the vagina over 5 contractions as if s/he were performing a manual rotation.

### The timing of the intervention

The intervention will begin once full dilatation has been diagnosed and the woman has the first urge to push or after one hour, whichever occurs first.

### Criteria for discontinuing or modifying the intervention

The intervention or sham will be discontinued if there is a clinical necessity or at the request of the participant. This could occur if there is evidence of fetal compromise necessitating emergent delivery or if the participant is in significant discomfort.

Each operator will complete a data collection form at the time of the procedure or sham, which will describe in detail what was done. Adherence with treatment allocation will be monitored by comparing these datasheets with the computer randomisation records.

All interventions and usual care provided by doctors and midwives looking after the participant will be allowed. However, if the doctor is intending to perform an operative delivery or a manual rotation, the woman will not be randomised. Data will be collected about use and timing of any manual rotations performed by the participant’s carers.

### Outcomes

#### Primary outcome

The primary outcome will be operative delivery (vacuum, forceps and/or caesarean section).

#### Secondary outcomes

Secondary outcomes will include the following:

1. Caesarean section (reported as proportion of participants who had a caesarean section) and
2. Serious maternal morbidity or mortality (combined outcome), which includes the following: post-partum haemorrhage requiring blood transfusion, third or fourth degree perineal trauma; dilatation and curettage for bleeding or retained placental tissue; cervical laceration; vertical uterine incision; vulvar or perineal haematoma; pneumonia; venous thromboembolism requiring anticoagulation; wound infection requiring hospital stay more than 7 days; readmission to hospital for obstetric-related causes; wound dehiscence; maternal fever of at least 38.5°C on two occasions at least 24 hours apart not including the first 24 hours; bladder, ureter or bowel injury requiring repair; genital-tract fistula; bowel obstruction; or admission to intensive care unit. This will be reported as a proportion of participants with serious morbidity or mortality.
3. Serious perinatal/neonatal morbidity or mortality within 6 weeks of birth (combined outcome), which will include the following: shoulder dystocia requiring manouvres other than McRoberts/suprapubic pressure or resulting in neonatal injury, 5-minute Apgars < 4; arterial cord pH <7.0 or lactate >10 or base excess < −15; seizures < 24 hours of age, intubation/ventilation >24 hours, tube feeding >4 days, admission to neonatal intensive care >4 days, neonatal jaundice requiring phototherapy, neonatal fracture, intraventricular/intracranial haemorrhage, subgaleal haemorrhage, neonatal blood transfusion, hypoxic ischaemic encephalopathy, or neuropraxia. This will be reported as a proportion of participants with serious morbidity or mortality.

#### Other outcomes

Other outcomes will be assessed during delivery admission and at t 6-weeks, 6-months, and 1-year postpartum.

The following outcomes will be assessed during delivery admission:

1. length of second stage (median)
2. time from intervention or sham until delivery (median)
3. estimated blood loss at delivery (median: visual estimation by midwife or doctor)
4. any perineal/vaginal trauma requiring suturing (proportion)
5. length of hospital stay (median)

The following outcomes will be assessed at 6 weeks:

1. still breast feeding (proportion)
2. satisfaction with birth (VAS scale) (median)
3. saw a health professional for depression since delivery (proportion)
4. health-related quality of life (SF-12) (median)

The following outcomes will be assessed at 6 months:

1. still breast feeding (proportion)
2. saw a health professional for depression since delivery (proportion)
3. health-related quality of life (SF-12) (median)

The following outcomes will be assessed at one year:

1. still breast feeding (proportion)
2. saw a health professional for depression since delivery (proportion)
3. health-related quality of life (SF-12) (median)
4. pelvic floor function (bowel, urinary, prolapse, and sexual function domains - using the Australian pelvic floor function questionnaire [34] (medians)

### Sample size

The sample size (254) was calculated on the basis of the primary outcome. The power calculation was based on our prospective cohort study of 160 women that was completed in May 2009 [3] and showed an operative delivery rate of 68% in the OP group, and from our survey of obstetricians conducted in 2010, who indicated they would perform a manual rotation for OP position if it reduced the rate of operative delivery from 68% to 50% [10]. To detect a reduction in the rate of operative delivery from 68% in the control group to 50% in the intervention group, a sample size of 127 women in each group (total = 254) will be required to have 80% power of finding a result. Alpha = 0.05 (2-tailed), Beta = 0.20 (Epi-Info version 3.3.2).

### Randomization/allocation concealment

Randomization will be stratified by parity, hospital site and epidural due to the potentially strong association between operative delivery (the primary outcome) and each of these factors. Randomization will be centrally controlled using computerized sequence generation, which can be accessed 24 hours per day using a toll-free telephone line.

In order to reduce the risk of randomising an ineligible participant, randomisation will occur immediately before the intervention or sham procedure is to be performed. An example of a participant becoming ineligible would be if the fetus rotated from the occiput posterior to occiput transverse position. Each investigator will complete a data collection form at the time the manual rotation or sham procedure is performed outlining the treatment allocation, clinical findings, and whether or not the fetus was successfully rotated.

### Blinding

The following groups will be masked:

1. The participants
2. The clinicians caring for the participant (including doctors and midwives)
3. The data collectors
4. The statisticians who will perform the analysis

### Unblinding

Unblinding will occur if the clinician requests it on the basis of clinical need or if the participant insists.

### Data collection, management and analysis

#### Study conduct

**Figure 1:**
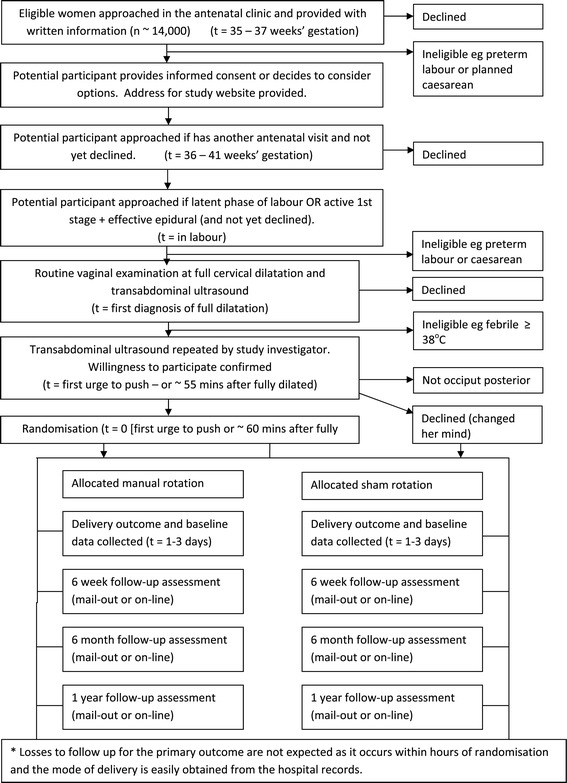
**An overview of the conduct of the POP-OUT Trial.**

Consent will occur at three possible time points Figure 1:

- Antenatally
- In the latent phase of labour
- In the active phase of the first stage of labour, with an effective epidural anaesthesia

An ultrasound will be performed at full dilatation by the clinician caring for the woman and the findings will be recorded on a data sheet immediately afterwards.

An hour after full dilatation or at the first urge to push, a study investigator (with no clinical responsibility for the woman in the trial) will confirm the OP position by a second (pre-procedure) bedside ultrasound. If the fetal position is still OP and the woman still wishes to participate, then the study investigator will randomise the woman to either manual or sham rotation. The treatment allocation will be recorded on a randomisation sheet that the investigator will keep on their person and not show to any of the participant’s carers.

After the manual rotation or sham has been performed the ultrasound will be repeated, ensuring that the woman and her carers do not see the screen. The investigator will leave and the woman will have her usual care from this point onwards. The investigator will record the findings of the vaginal examination he/she performed before the procedure, details of the procedure and post-procedure ultrasound findings on the same data sheet as the pre-procedure ultrasound. The study investigator will also keep this data sheet on their person and not show it to any of the participants’ carers.

### Consent

Participants will be provided with written information via information pamphlets, posters and the trial website. Informed consent will be obtained by research midwives or midwives/medical staff involved in potential participants’ care (Figure 1, Table 2). A detailed information sheet will be provided to all participants. Participants will be informed of the potential risks of manual rotation, including umbilical cord prolapse, given the opportunity to ask questions and informed that they have the right to change their mind at any time.

**Table 2:** **The POP-OUT study time-line for the schedule of enrolment, allocation and follow-up**

	**Enrolment**		**Allocation**		**Post-allocation**			
**Timepoint**	**35-37 wks gestation**	**1** ^**st**^ **stage labour**	**During 2nd stage of labour**	**Immediately after allocation**	**1-3 days**	**6 wks**	**6 mths**	**12 mths**
1^st^ Eligibility screen	X							
2^nd^ eligibility screen		X						
Informed consent	X	X						
Allocation			X					
Intervention				X				
**Assessments:**								
Labour and Delivery			X	X	X			
Operative delivery					X			
Perineal outcome					X			
Blood loss					X			
Maternal complications					X	X		
Hospital stay					X			
Readmission						X		
Neonatal outcomes					X	X		
NICU admission(s)					X	X		
Satisfaction with birth						X		
Breast feeding						X	X	X
Health related quality of life						X	X	X
Pelvic floor						X	X	X
Depression						X	X	X

### Primary outcome

Mode of delivery will be ascertained from the medical records.

### Other outcomes

Labour and delivery outcomes, perineal trauma, blood loss, duration of hospitalisation, short-term neonatal outcomes, and admission to the neonatal intensive care unit, maternal or neonatal readmission to the same institution, and other components of the combined secondary outcomes will be ascertained by a study investigator not involved in clinical care, using the medical records recorded contemporaneously by the clinician and by contacting the participants’ clinician for further information if required. Maternal depression, health related quality of life (SF-12), birth satisfaction (VAS), maternal or neonatal readmission to another institution, ongoing breast feeding, pelvic floor symptoms and components of the combined secondary outcomes will be collected by structured maternal questionnaires at 6 weeks, 6 months and 12 months post-delivery as outlined in section 15. Questionnaires will be completed by mail-out, online via the trial website and by telephone depending on the participants’ preferences (Figure 1, Table 2). Data collectors will be unaware of the treatment allocation at all times.

As the primary outcome is mode of delivery and randomisation occurs during the second stage of labour, we expect 100% ascertainment for the primary outcome.

Study investigators will perform site visits about four times per year to promote recruitment, provide education for clinical staff and site investigators and to audit centre medical records to verify the accuracy of the data collected by the sites.

Each participants will receive a phone call at each time point by research staff not involved in her care to ask her preference for follow-up. Unless she declines further participation, each participant will receive a reminder phone call and will be offered completion of the questionnaire by telephone if they feel they cannot complete it by mail or online.

### Data management

Data collected will be entered into a registered electronic database by research staff blinded to treatment allocation and who are not involved in the clinical care of the participants. Hardcopies of participants’ data will be stored in a locked office. The electronic database will include the study identification number but no directly identifying data such as medical record number, date of birth or personal address. The de-identified database will be backed up on a server at Royal Prince Alfred Hospital. Data linking identifying details to the study number will be kept at a separate location in a locked filing cabinet. At the end of the study, data will be kept in a locked filing cabinet, and de-identified electronic data will be kept on a portable medium such as a USB drive in a separate secure location at Royal Prince Alfred Hospital.

All electronic data will be checked for accuracy by a second member of the research team and any apparent data entry errors will be discussed by the primary investigators and investigated/corrected as required.

### Analysis

Analysis will be by intention-to-treat (according to treatment allocation), including withdrawals and losses to follow-up. Losses to follow-up for the primary outcome are not expected because randomisation will occur at full dilatation and the primary outcome is the mode of delivery.

The results will be reported according to CONSORT guidelines.

Demographics and other potential confounders will be compared by treatment allocation in a univariate analysis. Categorical outcome measures will be compared by proportions (chi-squared test), means for normally distributed data (*t*-test), or rank order for non-normally distributed data (Mann–Whitney-*U* test).

A logistic regression analysis of treatment allocation and other variables on the primary outcome measure, operative delivery, will be performed. The following variables will be considered for the logistic regression model: maternal body mass index, maternal age, maternal height, maternal ethnicity, gestation, induction of labour, gestational diabetes, neonatal gender, and RCOG CTG classification in the second stage of labour. Parity, study site and the presence of epidural for intrapartum analgesia at the time of randomisation will not be included because randomisation is stratified for these variables. Only variables where *P* <0.25 in the univariate regression will be included in the multivariate model. Continuous variables that do not show a linear association with the logit function will be divided into quartiles and treated as categorical. Interaction terms will be considered for treatment allocation versus each of the other variables and where clinically appropriate between non-treatment variables. *P* <0.01 will be considered evidence of interaction. Terms will be excluded from the model in a stepwise backward manner until all remaining terms are both statistically significant (*P* <0.05) and clinically significant (that is, removal of the term results in a clinically significant change in the estimate of the odds ratio of treatment allocation for the primary outcome). The analysis will be performed using SAS 9.2 (or a more recent version of SAS).

Subgroup analyses will be performed according to the technique of manual rotation employed (manual/whole hand versus digital/fingers) and according to operator ability (data will be divided into two approximately equal groups according to the success rate of the operator who performed the manual rotation).

### Data safety monitoring committee

Draft terms of reference for a data and safety monitoring committee provide for potential cessation of the trial if significant safety concerns are raised. The data and safety monitoring committee will consist of three people who are not involved in the study and do not have a working relationship with the primary investigators. Adverse events will be reported to the committee.

### Interim analysis and stopping rules

There will be no interim analysis. The Data Safety Monitoring Committee may advise that the trial be stopped if significant concerns about the safety of manual rotation are found.

### Harm

Any serious complications will be referred to the Data Monitoring Committee.

### Auditing

There will be no external auditing of the trial.

### Research ethics approval

This study has been approved by the Ethics Review Committee (RPAH Zone) of the Sydney Local Health District, Sydney, Australia, Protocol number X110410.

## Discussion

This trial addresses an important clinical question concerning a commonly used procedure that has the potential to reduce operative delivery and its associated complications. Due to the nature of the intervention, a number of issues are worthy of discussion.

First, empirical evidence suggests that blinding reduces bias in randomised controlled trials. However, blinding may be difficult in the case of procedural interventions. In this trial, we intend to assess the efficacy of blinding by asking the woman’s carer to guess the treatment allocation after manual rotation or sham rotation has occurred. The purpose of this is to allow the reader to assess the risk of bias associated with knowledge of treatment allocation.

Second, the efficacy of procedural interventions may depend on the experience and training of individual operators. The ‘success’ of manual rotation of individual operators will be assessed by recording the ultrasound determined fetal position after the manual rotation or sham procedure has been performed. We will report on any major differences between the success rates of individual practitioners.

Third, due to the ethics of consent in labour, consent will be obtained when it is unknown if the fetus will be in the occiput posterior position in the second stage of labour, which is an eligibility criterion. Thus, it is likely that only a minority of consented participants will be randomised, which will result in a large workload per randomisation (the pilot study was used as a reference).

Finally, women who progress rapidly in labour may give birth before they can be randomised and women with regional analgesia will have more opportunity to be consented. This could result in the study population having a higher background risk of the primary outcome than non-consented women who meet our eligibility criteria, which could impact the generalisability of our findings.

### Protocol amendments

If modification to the study protocol is considered necessary, then permission will be sought from the ethics committee and the changes will be described in the final report.

### Confidentiality

All the information collected from the study will be treated confidentially, and only the researchers will have access to it. Hard copies of data collection forms will be stored in a locked office. The electronic database will be de-identified and stored at a different location to codes linking identifying data to study identification numbers. The electronic database will be on Microsoft Access, password-protected, and only accessible by research staff.

### Roles and responsibilities

#### Trial management committee

The committee consists of Hala Phipps, Jon Hyett and Bradley de Vries, who are responsible for the following:

1. Study planning
2. Organisation of Steering Committee meetings
3. Randomisation
4. Reporting of any serious adverse events to the Data Monitoring Committee
5. Budget administration and organising contracts with individual centres
6. Providing advice for site investigators
7. Auditing and visiting sites
8. Data verification
9. Following up of study participants

### Site investigators

In each participating centre, a lead investigator (obstetrician) will be responsible for identification, recruitment data collection and completion of relevant trial forms, along with adherence with study protocol. Each lead investigator will be a steering committee member.

### Steering committee

The Steering Committee will be chaired by Brad de Vries, and all lead investigators will be steering committee members and are responsible for the following:

1. Recruitment of pregnant women on the study and liaising with principal investigators HP, JH and BD.
2. Reviewing progress of study and facilitating the smooth running of the trial.
3. Reporting the results of the trial.

### Data manager

The data manager will be responsible for maintenance of the trial IT system, data entry and data verification.

### Trial status

Start date: 16 April 2012.

Number currently recruited: 160.

## Abbreviations

ARM: artificial rupture of membranes
CS: caesarean section
NSW: New South Wales
NICU: neonatal intensive care unit
OA: occiput anterior
OP: occiput posterior
OT: occiput transverse
PPH: postpartum haemorrhage
RCT: randomised controlled trial
